# Early Diagnosis and Treatment of Pyoderma Gangrenosum: Reviewing Mobile Phone Photos Saved a Patient From Unnecessary Surgeries

**DOI:** 10.7759/cureus.54797

**Published:** 2024-02-23

**Authors:** Evangelos Keramidas, Stavroula Rodopoulou, Nikolaos Avgerinos

**Affiliations:** 1 Plastic Surgery, Kosmesis Aesthetic Plastic Surgery Center, Athens, GRC

**Keywords:** pyoderma gangrenosum after breast reduction, postoperative pyoderma gangrenosum, pyoderma gangrenosum (pg), pyoderma gangrenosum post surgery, pyoderma gangrenosum of the breast, diagnosis and treatment of pyoderma gangrenosum, diagnosis of pyoderma gangrenosum, breast ulcer, pyoderma gangrenosum

## Abstract

Pyoderma gangrenosum of the breast following surgery is a rare aseptic inflammatory cutaneous condition that causes very rapid progressing and expanding painful ulceration of the surgical site and the adjacent skin. The greatest issue concerning pyoderma gangrenosum is its diagnosis. Almost invariably, it is misdiagnosed as a wound infection, which results in delayed identification, lengthy antibiotic regimens, and ineffective detrimental surgical debridements, causing significant patient disfigurement. We present a rare case report of pyoderma gangrenosum complicating the surgical site of the breast reduction procedure two months after simultaneous performance of operations including breast reduction, abdominoplasty, and lumbar liposuction. The diagnosis was established within four hours from the initial lesion and symptom presentation due to the accurate evaluation of photographs sent from the patient’s mobile phone to the surgeon every half hour. Immediate appropriate treatment with oral corticosteroids within this time interval was initiated, resulting in favorable healing for the patient within four months.

## Introduction

Pyoderma gangrenosum is included among the dermatological complications that can affect plastic surgery procedures and is the most common and serious condition that can occur following surgery [1]. Its reported incidence has been found to be three cases per million per year in the USA [2,3]. Pyoderma gangrenosum is an aseptic inflammatory skin disease and therefore antibiotics and surgical debridement have no therapeutic evidence in its treatment, with the latter potentially further exacerbating and disseminating the disease to previously healthy tissues by the phenomenon of pathergy [4].

Its rarity and the fact that its clinical features and presentation resemble that of infection commonly misdirects surgeons leading to significant delays in its diagnosis. The median time from symptom presentation and correct diagnosis has been found to be 15 days, delaying in some cases even years [1,5].

Studies have shown the breast as the area most frequently associated with the occurrence of pyoderma gangrenosum following surgery [1,6] and breast reduction as the operation most frequently related to its appearance [1,5]. Usually, it appears on the breast surgical incision site four days to six weeks following surgery, as wound dehiscence rapidly progresses to expanding ulceration commonly accompanied by fever [1,7,8].

Laboratory images of pyoderma gangrenosum share similarities with sepsis-like syndrome with inflammatory markers being significantly elevated. Tissue biopsy revealing non-specific inflammatory infiltrate confirms the diagnosis of pyoderma gangrenosum, although early clinical suspicion is the key to initiating pertinent therapy and avoiding reoperation.

Treatment involves the immediate initiation of immunomodulatory medication such as corticosteroids or cyclosporine until ulcer healing, for a duration of approximately 4-5 months with gradual dose tapering, and observation for possible recurrences [1,9].

Pyoderma gangrenosum occurrence has been associated at a 70% rate with a number of systemic inflammatory diseases such as inflammatory bowel disease, autoimmune conditions, and malignancies [7,10]. Thus, investigation for such underlying pathologies is recommended upon establishment of diagnosis. However, in many cases, it has been noted in patients without a history of any predisposing factor [1].

We present the case of a 36-year-old female who underwent a combined operation of breast reduction, abdominoplasty, and lumbar liposuction, and two months after the procedures her left breast incision site presented a very rapidly progressing wound dehiscence and ulceration.

## Case presentation

A 36-year-old female patient was admitted on April 6, 2023, for a scheduled combined operation of breast reduction, abdominoplasty, and lumbar liposuction.

The patient’s medical history included a thyroidectomy in 2006 and a cesarean section in 2014. She was a non-smoker with no history of allergies, autoimmune diseases, or previous significant response to trauma or surgery. She weighed 68 kg, and her height was 169 cm.

The operation began with the patient under general anesthesia and breast reduction was performed first using the inverted T technique for reducing the volume and correcting the asymmetry of the breasts. About 224 g were resected from the right breast and 438 g from the left, respectively. Breast skin closure was performed using Monocryl 3-0 and 4-0 sutures.

Upon completion of breast reduction, an abdominoplasty procedure was carried out. The technique included the correction of a 4.5 cm rectus diastasis employing absorbable sutures, and at the end of the procedure, 430 g were resected from the right side of the abdomen and 380 g from the left side, respectively. No drains were placed in the breast or the abdomen at the end of the surgery.

The total duration of the combined procedures was six hours. The operating room temperature was set at 21 degrees Celsius for the prevention of hypothermia. Compression stockings along with sequential compression devices were used during the procedure as preventive methods for deep venous thromboembolism. Preoperatively, the patient received s.c. tinzaparin sodium and a single dose of IV vancomycin and piperacillin/tazobactam were administered 30 min before the induction of anesthesia.

The patient’s postoperative course was uneventful and on the second postoperative day. She was discharged from the hospital with a prescription for a subcutaneous anticoagulant medication for 12 days and returned to her city.

However, two months postoperatively, at 12:00 a.m. on May 27, the patient called the senior author informing that she was experiencing wound dehiscence at the T junction of breast reduction on her left breast, as well as a fever of 38 degrees Celsius. The surgeon requested photographic images of the lesion through the Viber mobile application owing to the remote location of the patient.

**Figure 1:**
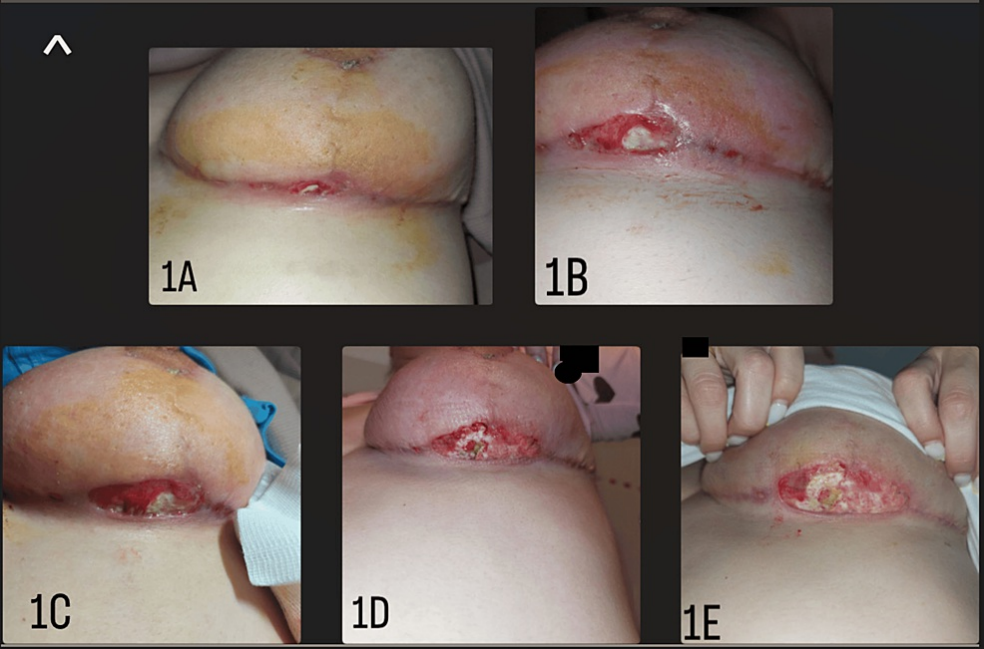
Photographs of the patient's left breast captured with a mobile phone camera (A) 12:00 p.m., (B) 14:00 p.m., (C) 14:30 p.m., (D) 15:00 p.m., and (E) 15:30 p.m. show the rapid expansion and progression of the ulcer doubling, even tripling in size in a very short period of time within four hours after initial ulcer appearance.

**Figure 2:**
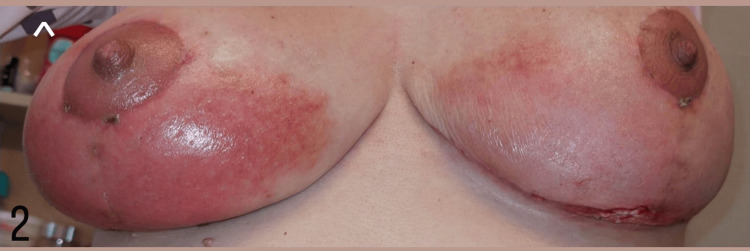
Photograph of the patient taken three hours after the initial symptoms appeared Skin erythema and swelling indicate impending involvement of the right breast as well. Note that the nipple-areola complex remains unaffected in both breasts.

At 14:00 p.m., the patient called and mentioned the expansion of the wound, which had doubled in size. At that time, the surgeon asked her to send pictures every half hour including both breasts. After a series of five photographs (Figure 1), the senior author noticed a very extensive deterioration of the wound and an erythematous rash expanding on the skin of the other breast (Figure 2).

Four hours after the initial phone call to the patient, due to the very rapid expansion of the wound, the senior author suspected pyoderma gangrenosum and suggested oral administration of methylprednisolone 32 mg/day (0.5 mg/kg/day) and ciprofloxacin 500 mg twice a day. He also advised obtaining a specimen from the lesion for microbe culture. Additionally, care of the open wound with a local antiseptic was recommended, and the patient was instructed to travel to Athens.

The patient executed all the recommended instructions immediately, and three hours later, arrived in Athens.

In our clinic in Athens, the senior author noticed bilateral breast skin erythema and swelling, minimal serpiginous drainage, and extensive ulceration of the left breast incision site. However, the nipple-areolar complex was unaffected in both breasts. Compared with the photographs, no further lesion expansion was observed. A specimen for microbiological culture was obtained along with two incisional biopsies for histopathology examination, one from the center of the lesion of the open wound and one from the borders of the ulcerative lesion [1]. Body temperature measurement documented 38 degrees Celsius fever and laboratory workup revealed leukocytosis (18,000 cells/μL WBC) as well as elevated C-reactive protein (4 mg/dl). Subsequently, the patient was referred to a dermatologist who agreed with the initial diagnosis and dosage of methylprednisolone as was initially administered by the surgeon, adding only an antibiotic-containing ointment applied locally inside the ulcerated lesion.

**Figure 3:**
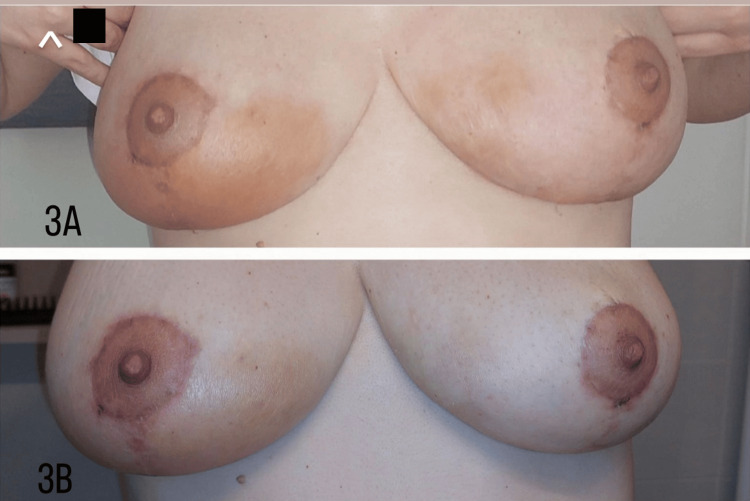
Patient photographs taken (A) two days and (B) five days after initiating oral corticosteroid medication, showing dramatic improvement with subsidence of the erythematous rash and edema in both breasts

During the next 24 hours, a response to the therapy was noticed, characterized by swift improvement with regard to the erythematous rash on the right breast also on the left breast, but not regarding the ulceration of the left breast (Figure 3).

About 48 hours after the initiation of oral methylprednisolone, laboratory workup revealed significant improvement in the WBCs and C-reactive protein values. No other ulceration developed in the following days, and the sites of abdominoplasty surgery remained unaffected the whole time.

**Figure 4:**
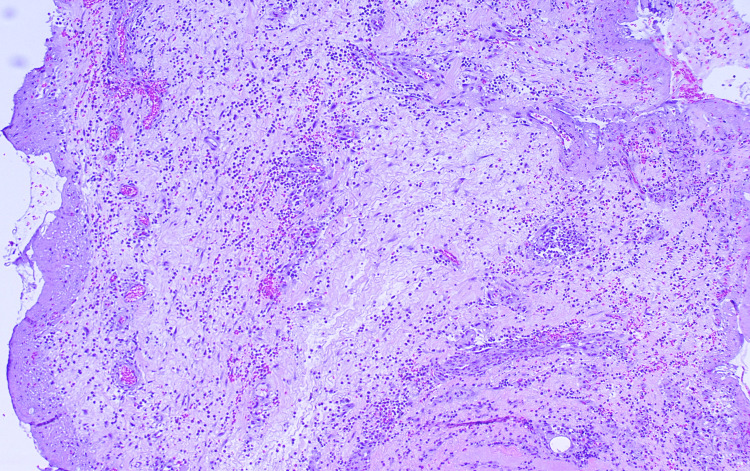
Histological findings display an intense and diffuse neutrophilic infiltrate, consistent with the diagnosis of pyoderma gangrenosum

Two days later, histopathology revealed in both specimens intense acute inflammatory infiltration of the reticular dermis with predominantly polymorphonuclear neutrophil leukocytes and the distribution of them in the underlying subcutaneous fat (Figure 4).

**Figure 5:**
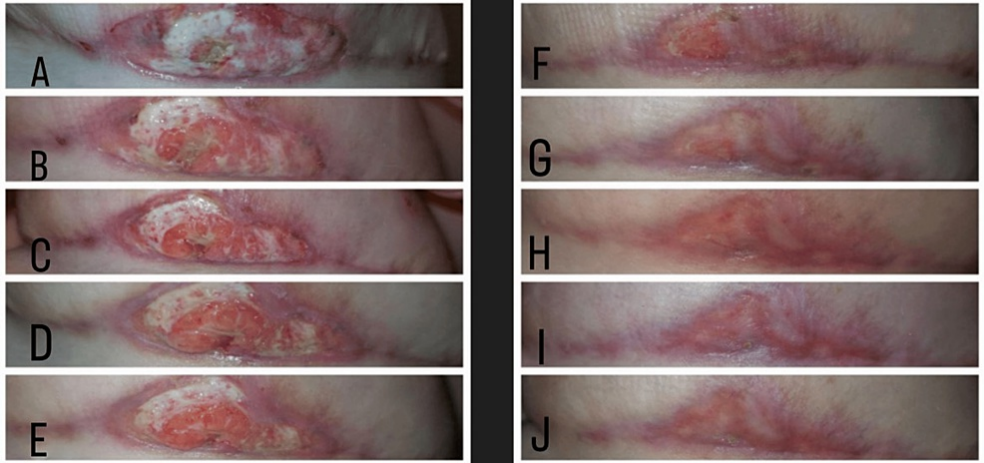
Series of patient photographs showing the progression of a left breast ulcer toward healing under treatment with oral methylprednisolone medication Left from top to bottom (A)-(E): Day one to day five under treatment; right from top to bottom (F)-(J): Images each day after the first month with treatment.

The results of both the microbiological cultures were negative. Histopathology confirmed the initial diagnosis of pyoderma gangrenosum, leading us to double the oral methylprednisolone dosage from 32 mg to 64 mg per day (1 mg/kg/day) (Figure 5).

**Figure 6:**
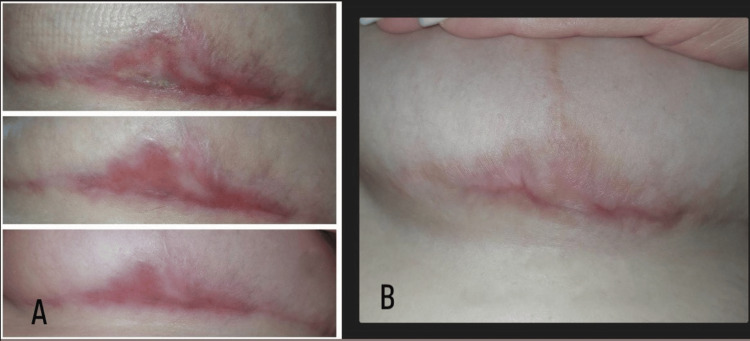
Comparison of wound healing (A) 3 1/2 months and (B) 4 months after the onset of symptoms, showing excellent healing

Four months later the ulcer had completely healed and oral methylprednisolone medication was gradually tapered (Figure 6). However, we remain vigilant for any possible recurrence.

Follow-up during treatment with corticosteroids included assessment for systemic side effects associated with their use [4].

In the interim, all possible investigations concerning autoimmune conditions as well as malignancies that have been associated in the literature with the appearance of pyoderma gangrenosum were performed [1,4-7,10]. All results were negative.

## Discussion

Pyoderma gangrenosum is a very rare non-infectious, aseptic inflammatory disease, potentially destructive if not early diagnosed [11], and appears most frequently on the breast [1,4-8,10].

Five subtypes of pyoderma gangrenosum have been described in the literature namely ulcerative, bullous, vegetative, and peristomal [4]; however, surgery and the tissue trauma it induces has been considered as triggering factor possibly inciting a response called pathergy [9], giving rise to a separate variant called post-surgical pyoderma gangrenosum [2,8].

Clinically pyoderma gangrenosum following surgery presents as wound dehiscence of the surgical site rapidly enlarging to painful ulceration [8].

Breast reduction surgery is the procedure most frequently associated with its occurrence, involving the breasts in a bilateral, almost symmetrical manner, characteristically leaving the nipple-areolar complex unaffected during the course of the disease [1,5-7] which is considered a pathognomonic feature for the disease [8]. Pyoderma gangrenosum of the breast has been reported to develop as early as four days to six weeks following breast surgery [5,7,8]. However, a case in which pyoderma gangrenosum complicated the breast reduction surgical site even after seven years has been described [12,13].

Studies have found that the median time for correct diagnosis and proper treatment initiation after presentation was 15 days, and in some cases, it lasted even years [1,4,5,7]. To facilitate recognition, aiming to reduce the errors in diagnosis due to its similarity with other ulcerating conditions, In 2004 Su et al. published diagnostic criteria for pyoderma gangrenosum [10]. Major criteria include the rapid progression of a painful ulcer with irregular, undermined violaceus borders and the exclusion of other ulcer etiology usually through biopsy [10]. More recently in 2018 Maverakis et al. proposed criteria for the diagnosis of pyoderma gangrenosum removing the component of exclusion of other ulcer etiology from the major criteria [14].

In our case, early diagnosis four hours after the onset of symptoms saved the patient from surgical debridements, prevented ulcer appearance on the other breast, and resulted in early restriction and healing of the ulcer. The combination of high-definition photographs along with high clinical suspicion of this very rare disease ensured a very early diagnosis within four hours. Because of this, as well as excellent patient cooperation, she was saved from any further operations and was never hospitalized.

In our case, the patient’s photographs were the decisive element leading the senior author to consider pyoderma gangrenosum, and that was because they made evident the very rapid deterioration of the ulcerated wound, which could not be justified simply by an infection. The surgeon considers that in the absence of a photographic documentation aid, based only on the description of the patient the diagnosis probably would have been delayed. The review of all photographs, particularly after the first three hours was highly indicative of pyoderma gangrenosum.

Every surgeon involved in breast surgery regardless it is reconstructive or aesthetic should remain cognizant of pyoderma gangrenosum and consider it in the diagnosis of breast ulcers following surgery.

Whenever we encounter an accelerating lysis of the skin, doubling or even tripling in its size within a few days and noteworthy, within hours like in our case, especially in a breast that has undergone surgery, pyoderma gangrenosum must be suspected. The diagnostic modality should involve obtaining a culture specimen for microbiology to exclude bacterial cause of ulceration, as well as an incisional biopsy both from the center of the lesion and from the borders of the ulcer, ensuring the specimen includes deep reticular dermis and reaches the subcutaneous fat [4,7,10]. Laboratory work-up including a complete blood count, and inflammatory markers namely C-reactive protein and erythrocyte sedimentation rate as well as liver and renal function tests is also necessary [9].

The therapeutic approach includes the administration of oral systemic corticosteroid medication (0.5-1 mg/kg/day) for its anti-inflammatory properties [4,9] and local wound care measures aiming to reduce the risk of secondary infection, which commonly occurs in pyoderma gangrenosum wounds [7]. A local antibiotic ointment applied on the ulcerated wound as well as an oral broad-spectrum antibiotic covering skin flora are effective in bacterial super-infection prevention [7]. Additionally, local antibiotic creams combined with moist occlusive dressings, or the use of moist antibacterial dressings, both assist in wound desiccation prevention [7]. Due to the pathergy phenomenon a surgical intervention on the affected site and the subsequent trauma it induces will worsen the lesions and will further spread the disease to previously healthy, unaffected tissue, therefore should be avoided [4].

Response to treatment manifests with significant reduction of the skin erythema and pain as well as inflammatory markers within 24-72 hours upon treatment initiation with corticosteroids [3,4,10] and should last until complete ulcer healing [8].

## Conclusions

We present a case in which a diagnosis of pyoderma gangrenosum was established very early, within hours from the initial ulcer appearance on the surgical site of the breast reduction operation. The value of our case lies in the fact that the diagnosis was established very quickly, with significant help from the pictures sent by the patient, and pertinent medication was immediately initiated. Consequently, favorable healing was attained and no disfiguring scar resulted. In contrast to other cases presented in the literature, the patient did not undergo any further surgery.
